# Congenital biscuspid aortic valve in pediatric and early adulthood: Is there a relationship between the valvular leaflet fusion pattern and other functional parameters

**DOI:** 10.1186/1532-429X-18-S1-P344

**Published:** 2016-01-27

**Authors:** Noha Behairy, Ahmed Ramadan, Ahmed E Kharabish

**Affiliations:** Radiology, Cairo University, Cairo, Egypt

## Background

Bicuspid aortic valve (BAV) represents the most frequent congenital cardiac abnormality resulting in premature valvular degeneration and aortic dilatation.

Our aim was to detect a relationship between the leaflet fusion pattern and other functional parameters including valvular regurge, stenosis and pressure gradient.

## Methods

One hundered patients between 3 months to 26 years were included in the study. They were 78 males and 22 females. BSA for the patients ranged from 0.45-2.27. All patients were subjected to clinical examination, transthoracic echocardiography and CMR on a 1.5T machine. We recorded the leaflet fusion pattern, presence of AS, AR, pressure gradient, EF, LVEDV, aortic diameter at the annulus, sinus, arch and ascending aorta levels. Associated findings were all recorded.

## Results

Sixty patients had right and left coronary cusps (R-L) fusion showing mean pressure gradient of 23.5 ± 14.8 of those 48% patients showed AS while 52% had AR. Forty patients had right and noncoronary cusps (R-N) fusion with 44.5 ± 31 pressure gradient with P = 0.02, of those 75% had AS while 45% had AR with 20% showing combined lesions. Ejection fraction was within normal range except in patients with associated myocardial lesions. LVEDV ranged from 49-185 ml. Aorta was dilated in 38 cases with no predilection for any leafleted fusion type. Associated co-aorctation of the aorta was detected in 22 patients of which18 had R-L fusion. Other associations were PDA (8 cases), VSD (8cases), hypoplastic aortic arch (6 cases), DCM and DORV (2 cases each).

## Conclusions

Our study showed that patients with R-L leaftet fusion had lower pressure gradient and a higher association with co-aorctation of the aorta, while patients with R-N leaflet fusion had higher pressure gradient with higher incidence of aortic stenosis.

**Figure 1:**
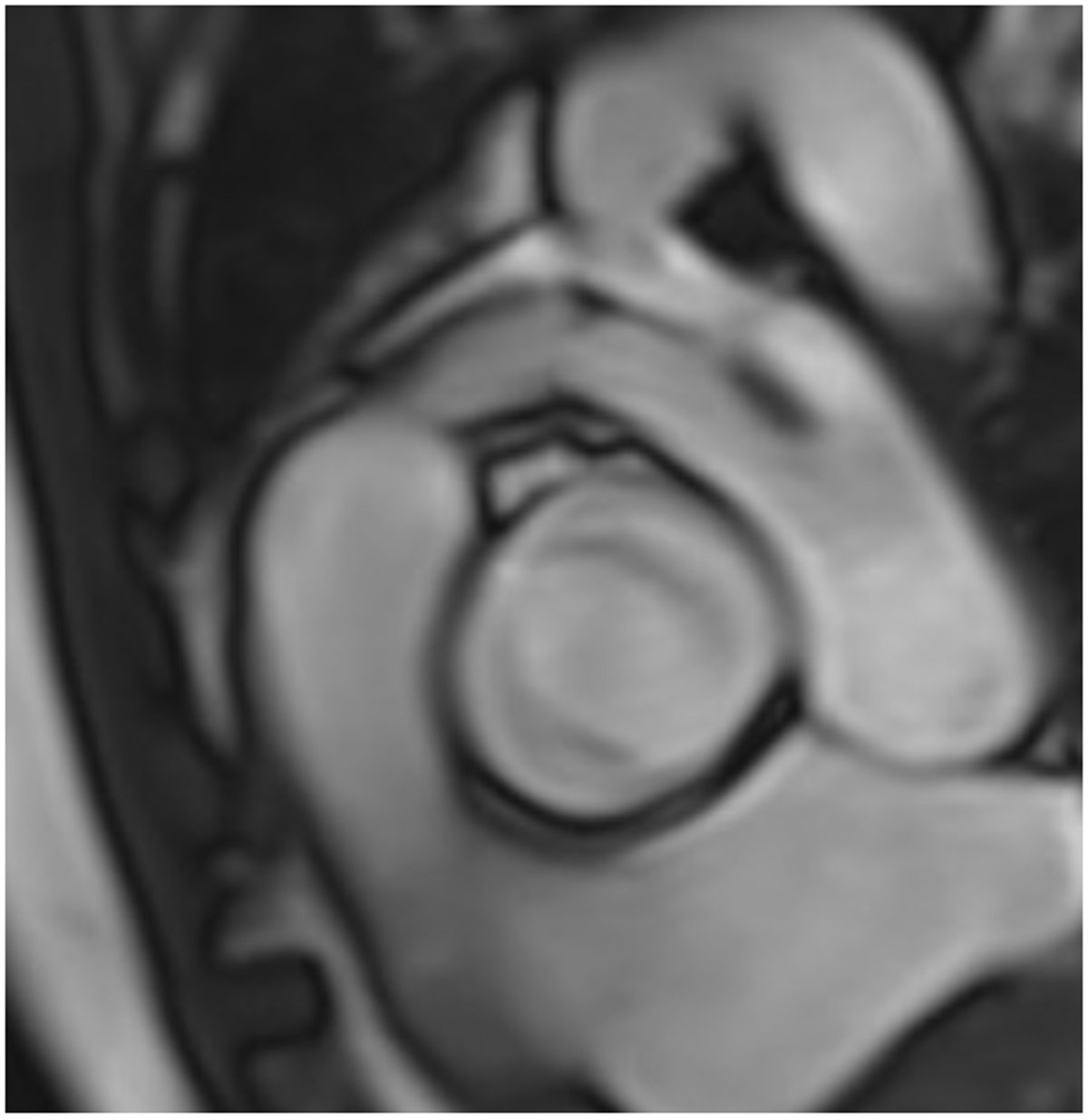
**bicuspid aortic valve in a 10 yr old male**.

